# Intestinal mucosal barrier injury: insights from interdisciplinary perspectives and therapeutic approaches

**DOI:** 10.3389/fnut.2026.1703567

**Published:** 2026-03-24

**Authors:** Zhi Wang, Nuo Li, Chen Zhao, Chenjing Hui, Sen Zhao, Qi Zheng, Hanwen Cheng, Yongcheng Ding, Bei Miao

**Affiliations:** 1 Department of Gastroenterology, The Affiliated Hospital of Xuzhou Medical University, Xuzhou, Jiangsu, China; 2 The First Clinical Medical College, Xuzhou Medical University, Xuzhou, Jiangsu, China; 3 The Second Affiliated Hospital of Xuzhou Medical University, Xuzhou, Jiangsu, China; 4 Department of Traditional Chinese Medicine, XuZhou Central Hospital Affiliated to Medical School of Southeast University, Xuzhou, Jiangsu, China; 5 The Affiliated Xuzhou Municipal Hospital of Xuzhou Medical University, Xuzhou, Jiangsu, China; 6 Department of Psychiatry, The Affiliated Xuzhou Oriental Hospital of Xuzhou Medical University, Xuzhou, Jiangsu, China; 7 Shanghai Academy of Environmental Sciences, Shanghai, China

**Keywords:** diverse perspectives, integrated therapeutic approaches, intestinal mucosal barrier, intestinal mucosal barrier injury, multidisciplinary synthesis

## Abstract

The intestinal mucosal barrier serves as a critical defense system essential for nutrient absorption, maintenance of intestinal microecology, and immune homeostasis. Growing evidence from clinical and interdisciplinary studies has established intestinal mucosal barrier injury as a complex and multifactorial condition, drawing research interest across numerous scientific fields. This review provides a multidisciplinary synthesis, integrating insights from Traditional Chinese Medicine (TCM), Nutrition, Environmental science, Psychology, Genetics and Food science to deconstruct its complex pathophysiology. We systematically consolidate these diverse perspectives to elucidate disease mechanisms, clinical manifestations, and detection strategies. Moreover, we propose a comprehensive framework to guide future research and inform the development of effective, integrated therapeutic approaches.

## 1 Introduction

The intestinal mucosal barrier serves as a critical defense system, safeguarding the gastrointestinal tract against harmful pathogens and toxins. Beyond this protective role, it is essential for regulating nutrient absorption, maintaining immune homeostasis, and preserving the maintenance of intestinal microecology. This barrier is composed of a dynamic and complex network, including mechanical, chemical, biological, and immune barriers, which work in concert to maintain intestinal and systemic health (1). When the integrity of this barrier is compromised, it can lead to a cascade of detrimental effects extending well beyond the gastrointestinal tract, impacting multiple organ systems.

In recent years, studies have confirmed that intestinal mucosal barrier injury is closely related to the occurrence and development of various diseases in multiple human systems, such as inflammatory bowel disease (IBD) (2), liver cirrhosis (3), obesity (4, 5), diabetes (5, 6), and traumatic brain injury (7, 8). Given these extensive connections, in-depth exploration of causes, clinical manifestations, and detection methods of intestinal mucosal barrier injury holds significant clinical relevance. It helps reveal potential connections between multiple systems and disciplines and develop early prevention and treatment strategies.

This study systematically examines current research on intestinal mucosal barrier injury through an integrated, multi-perspective analytical framework. It further clarifies the potential associations between intestinal mucosal barrier injury and different systems as well as different disciplines. We hope that the comprehensive framework proposed in this review can stimulate interdisciplinary research and promote innovative development in related fields. The article is structured as follows: (1) An overview of the intestinal mucosal barrier; (2) Clinical manifestations and detection methods; (3) Therapeutic framework from a multi-system perspective; (4) Research status from a multi-disciplinary perspective; (5) Prospects for intestinal mucosal barrier injury.

## 2 Review methods

### 2.1 Literature search

This systematic review was carried out according to the Preferred Reporting Items for Systematic Reviews and Meta-Analyses (PRISMA) guidelines. Comprehensive literature searches were performed in PubMed, Web of Science, Scopus and Google Scholar up to August 2025, mostly in the past 5 years. The search strategy combined keywords related to “intestinal mucosal barrier injury”, “human health”, “human systems”, “detection methods” and “multiple disciplines”, including: (intestinal mucosal barrier OR intestinal mucosal barrier injury) AND (human health OR multiple systems OR digestive system OR urinary system OR nervous system OR respiratory system OR integumentary system) AND (detection methods OR direct detection OR indirect detection) AND (traditional Chinese medicine OR nutrition OR environmental science OR psychology OR genetics OR food science). No language and nation restrictions were applied. Additionally, reference lists of eligible studies were manually screened (snowballing approach) to identify supplementary literature.

### 2.2 Study selection

We read and checked the eligibility of the articles based on title and abstract on the website, then assessed the full text if applicable. Under the same conditions, we prioritized selecting articles with higher influence. We then list the selected articles in a table, extracted valid and key information, and finally started writing the text according to the prepared outline.

## 3 An overview of the intestinal mucosal barrier

### 3.1 Composition of intestinal mucosal barrier

The intestinal mucosal barrier is mainly composed of mechanical barrier, chemical barrier, biological barrier and immune barrier.

Firstly, the integrity of the mechanical barrier depends on intestinal mucosal epithelial cells and the connecting proteins between them. Tightly junction proteins (such as Occludin proteins and Claudin proteins), firmly link these components together and form a physical barrier that effectively prevents the entry of harmful substances into the body through the cellular gap (9).

Secondly, the chemical barrier is mainly composed of substances secreted by the epithelial cells of the intestinal mucosa (such as digestive juice and mucins) and secretions of the intestinal flora, such as SCFAs. The mucus layer covers the surface of the intestinal mucosa and forms a protective barrier (10).

Further, biological barriers are the microbiota that are normally parasitized in the gut, including intestinal probiotics and intestinal pathogenic bacteria. These microbiota exert colonization resistance against foreign strains by occupying intestinal ecological niches and producing antibacterial substances, thereby maintaining the homeostasis of the intestine (11).

Finally, the immune barrier is primarily composed of the gut-associated lymphoid tissue (GALT), which forms an integrated network of inductive and effector sites. Key components include Peyer’s patches, isolated lymphoid follicles, and mesenteric lymph nodes. A critical function of this barrier is immune exclusion, mediated by the secretion of secretory IgA (sIgA) into the gut lumen. These sIgA antibodies can bind to food antigens forming complexes that are subsequently cleared by peristalsis. At the same time, antigen- presenting cells in the intestinal mucosa, such as dendritic cells (DCs) and macrophages, are able to recognize harmful substances that invade the body. They work together to remove these harmful substances and maintain the immune function of the intestines^12^.

### 3.2 Mechanisms of intestinal mucosal barrier injury

The mechanisms of intestinal mucosal barrier damagemainly include ischemia–reperfusion injury, inflammatory response, infection, and drug injury.

In pathological conditions such as shock, severe burns, and acute pancreatitis, the intestine may suffer from different degrees of ischemia–reperfusion injury (13, 14). The initial ischemic phase induces structural alterations in the intestinal mucosal epithelium, compromises barrier integrity, and disrupts cellular metabolism. Subsequent reperfusion triggers a burst of reactive oxygen species (ROS). This oxidative damage impairs intestinal epithelial cells and tight junctions, further deteriorating mucosal barrier function and amplifying the inflammatory response (15). They may also cause severe damage to distant parts of the body (16).

An excessive inflammatory response can lead to tissue damage. In these conditions, activated immune cells release a cascade of cytokines and inflammatory mediators. These substances can destroy intestinal mucosal epithelial cells and their tight junctions while defending against pathogens, leading to impaired intestinal mucosal barrier function (17). For example, the dysregulation of autophagy process leads to the upregulation of Claudin-2. The increase in Claudin-2 will decrease the tightness of intestinal epithelial junctions, increase the intestinal permeability, overactivate the immune system, and ultimately cause inappropriate inflammation (18, 19).

In addition, infection is an important factor. During localized gut infections, pathogens can subvert epithelial signaling pathways and damage tight junctions via both paracellular and transcellular mechanisms. The disruption of the barrier will result in the passive passage of commensal bacteria, microorganisms, endotoxins and proteases through the epithelium, which in turn triggers systemic inflammatory reactions and establishes a vicious cycle of damage and inflammation (20). Representative infectious agents known to impair intestinal tissues include Shiga toxin-producing bacteria (21, 22), *Candida albicans* (23), and so on.

**Figure 1:**
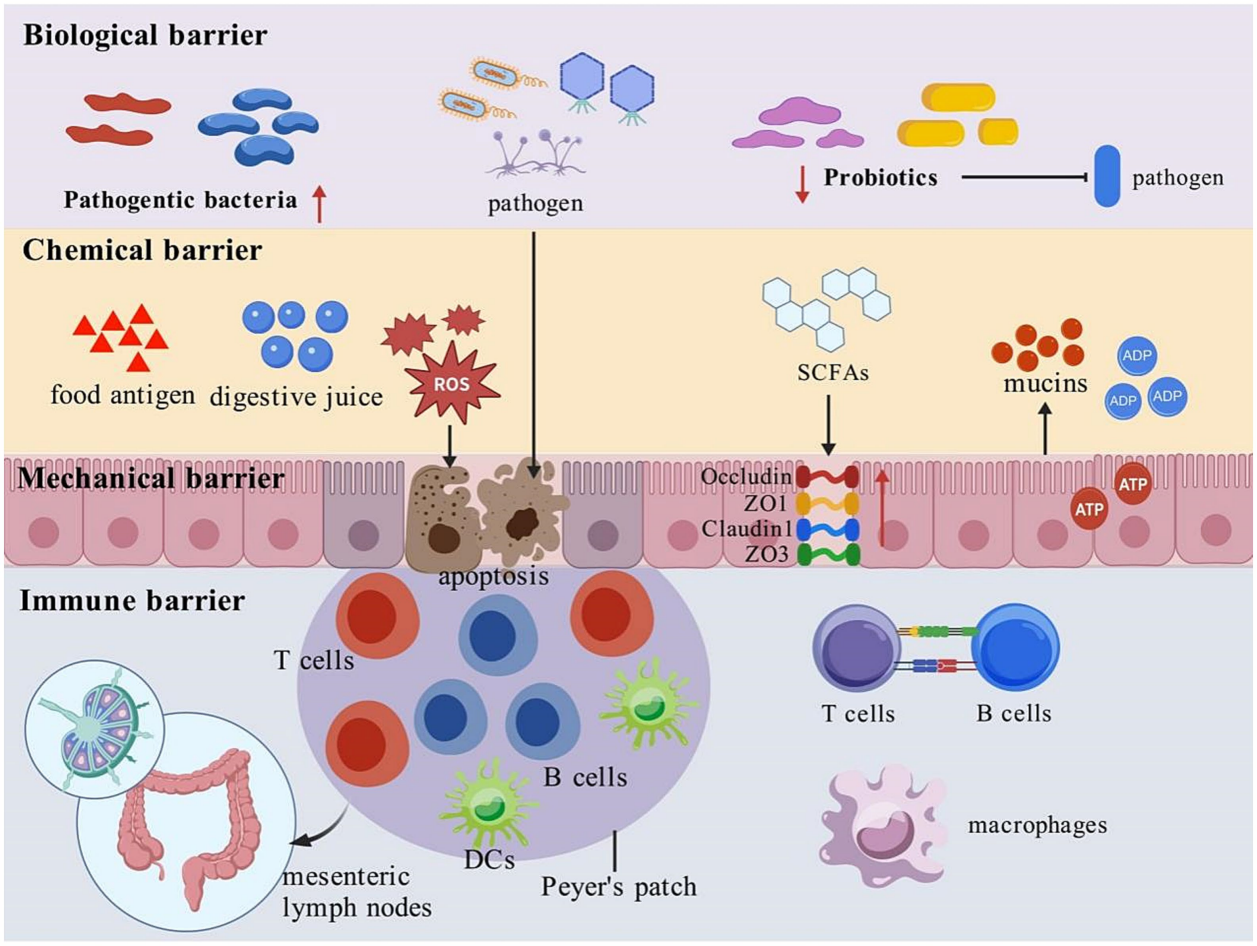
Composition of intestinal mucosal barrier and mechanisms of intestinal mucosal barrier injury. This figure shows the four major barriers of the intestinal mucosal, namely the biological barrier, chemical barrier, mechanical barrier and immune barrier. In the biological barrier, the number of pathogenic bacteria tends to increase and pathogentic bacteria tends to decrease against pathogens invasion. The chemical barrier involves substances such as food antigens, digestive juice, and mucins to play a defensive role. The mechanical barrier maintains structural integrity through tight junction proteins (such as Occludin, ZO1, Claudin1, ZO3). And the immune barrier involves various immune cells such as T cells, B cells, macrophages, DCs, and GALT. Together, these elements interact to prevent pathogen invasion and preserve intestinal integrity. SCFAs, short-chain fatty acids; ATP, adenosine triphosphate; ADP, adenosine diphosphate; DCs, dendritic cells; ROS, reactive oxygen species; ZO-1, Zonula Occludens-1; ZO-3, Zonula Occludens-3.

Notably, certain drugs including antibiotics (24) and NSAIDs (25) can directly damage intestinal epithelial cells and tight junctions. Their long-term use also disrupts the gut microbiota, thereby compromising both chemical and biological barriers and creating a vicious cycle of mucosal injury (26). The illustrations corresponding to the content in Sections 2.1 and 2.2 can be found in Figure 1.

## 4 Clinical manifestations and detection methods

### 4.1 Clinical manifestations

The clinical manifestations of intestinal mucosal barrier injury are diverse and complex, ranging from mild indigestion to severe IBD. Clinically, patients may experience symptoms such as abdominal pain, diarrhea, and constipation. Moreover, intestinal mucosal barrier injury may lead to increased intestinal permeability, making it easier for pathogens and harmful substances to enter the blood circulation and trigger systemic inflammatory responses (27). In some cases, intestinal mucosal barrier injury is also associated with a lack of dietary fiber, which may increase susceptibility to pathogens, thus causing long - term health problems (28). As a result, it is crucial to gain an in-depth understanding and make timely identification of these clinical manifestations for early intervention and treatment.

### 4.2 Detection methods

#### 4.2.1 Direct detection

Direct detection is used to assess intestinal mucosal barrier function by observing histologic manifestations of the intestinal mucosa and measuring intestinal mucosal permeability and analyzing tight junction protein expression.

The histological observation of the intestinal mucosa under light and electron microscopy can visually demonstrate the pathological changes in the organization of the intestinal mucosal epithelium. For instance, Nicholas Baidoo et al. reported a reduction in goblet cell numbers and degenerative changes in mucin density in the colon of elderly individuals compared to younger subjects (29). While this method is straightforward to implement, its interpretation can be influenced by subjective factors, such as variability in tissue processing, staining intensity, and the evaluator’s criteria for defining pathological changes, potentially affecting the reproducibility of results.

The intestinal mucosal permeability is an important indicator for evaluating the function of the intestinal mucosal barrier (30). Common clinical and experimental approaches include the lactulose/mannitol test and measurement of circulating D-lactate levels. These methods assess the changes in intestinal mucosal permeability by detecting the absorption and excretion status of specific substances in the intestine. Furthermore, these methods generally provide an indirect and global assessment of permeability, lacking the spatial resolution to pinpoint the specific site of barrier breach within the gastrointestinal tract. Consequently, results must be interpreted with caution and in conjunction with other clinical findings.

The integrity of the intestinal mucosal barrier function can be assessed by measuring the expression and distribution of tight junction proteins, such as ZO-1 (31) and Occludin (32). Researchers mainly analyze the expression of tight junction proteins by protein blotting and confocal laser scanning microscopy, and use RNA sequencing and bioinformatics for signaling pathway analysis (33). However, these approaches provide a static snapshot of barrier status at a specific time and from a specific sample site, failing to capture the dynamic, real-time functional state of the entire intestinal barrier in a living organism.

#### 4.2.2 Indirect detection

The commonly used indirect detection methods encompass bacteriologic examination, endotoxin and antibody testing, as well as testing for infection-related biomarkers.

Bacteriologic testing is a method used to detect intestinal bacterial translocation. By collecting specimens such as peripheral blood and peritoneal fluid for bacterial culture, it is possible to clarify whether intestinal bacterial translocation has occurred. However, traditional culture methods suffer from time-consuming and low positive rates. Recent studies indicate that during colitis, host cells can sense gut microbiota disturbances and participate in defense mechanisms. Absolute quantification techniques using multiple reaction monitoring have confirmed the presence of a variety of bacterial peptides in plasma and serum, most of which are associated with the bacterial composition of the small intestine. Their physicochemical properties may enable them to selectively cross the intestinal mucosal barrier and resist fibrinolysis (34).

Endotoxin is a product of bacterial metabolism and lysis. When intestinal bacteria are translocated, endotoxin can enter the circulation through the damaged intestinal mucosa. Therefore, the degree of damage to the barrier function of the intestinal mucosa can be indirectly assessed by detecting endotoxin and its antibody levels in the blood (35). However, this method lacks specificity, as elevated circulating endotoxin can originate from any systemic Gram-negative bacterial infection, not exclusively from intestinal translocation.

In contrast, infection-related biomarkers such as soluble myeloid expression-triggered receptor (sTREM-1), C-reactive protein, and procalcitonin can reflect the infection status of the organism. These biomarkers also indicate the degree of inflammatory response, so the impairment of the intestinal mucosal injury function can be indirectly assessed. For example, the ratio of T1 and T2 and the levels of related cytokines in the tissues of patients with IBD were detected to explore the pathogenesis (36). In addition, abnormal NOX1-NOS2 activity is a hallmark of ileitis (37). However, a single measurement provides limited clinical value, and serial monitoring is required to reveal trend dynamics.

In both clinical practice and scientific research, it is recommended to adopt a combined application of multiple detection methods, establish a personalized dynamic monitoring system, and conduct comprehensive analysis by integrating clinical manifestations with other auxiliary examination findings. These systematic improvements will collectively enhance the accuracy of intestinal mucosal barrier function assessment and its clinical utility.

**Figure 2:**
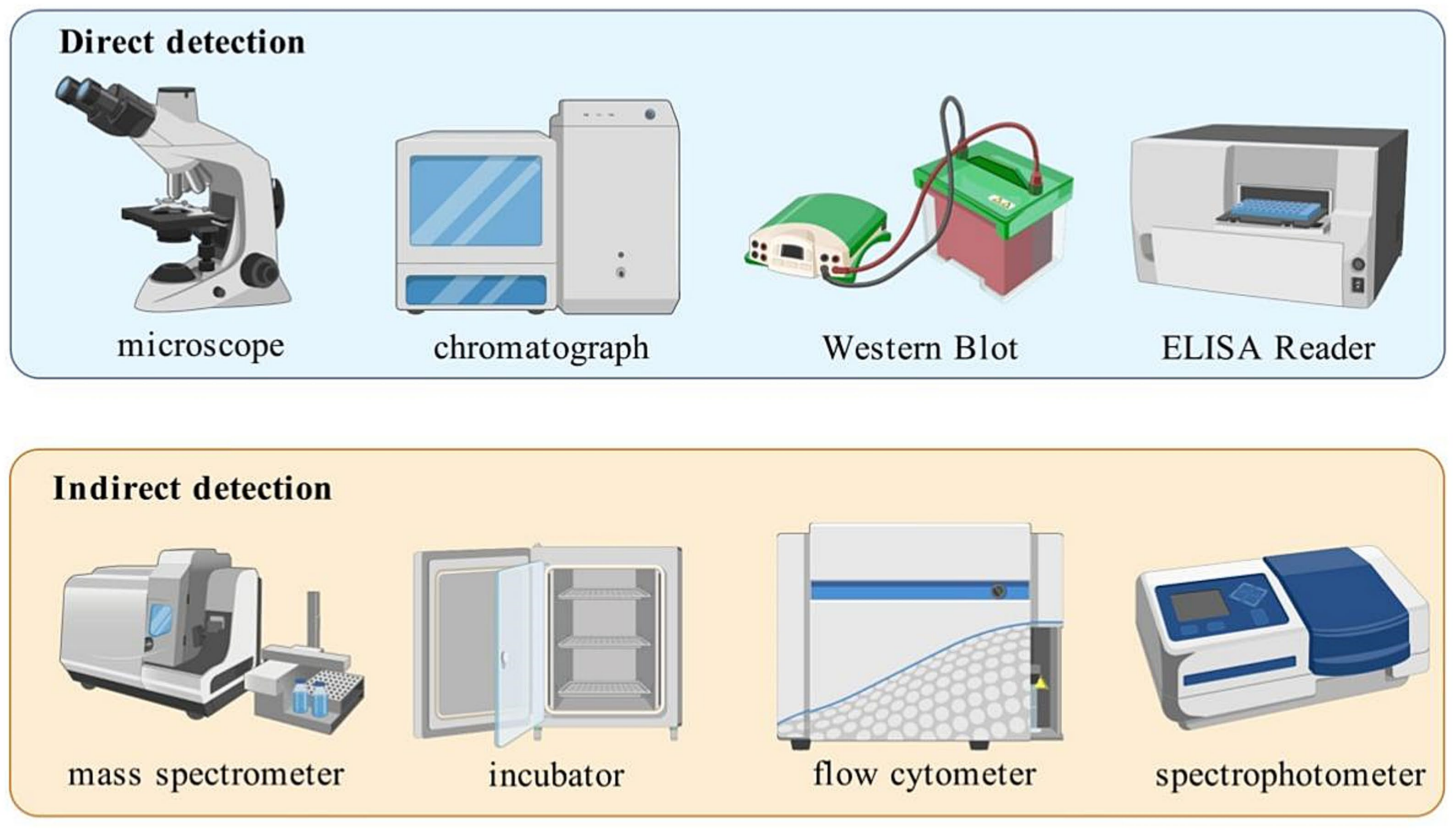
Detection methods of intestinal mucosal barrier injury. The first row illustrates the main direct detection instruments for intestinal mucosal barrier damage, including microscope, chromatograph, Western Blot and ELISA Reader. The second row presents the main indirect detection instruments, including mass spectrometer, incubator, flow cytometer and spectrophotometer.

The illustrations corresponding to the content in Sections 3.2 can be found in Figure 2 and Table 1.

**Table 1:** Comparison of advantages and disadvantages of detection methods.

Detection methods		Instrument	Advantages	Disadvantages	Reference
Direct detection	Histological observation	Light microscopy, Electron microscopy	Direct pathological evidence, Gold standard for etiological diagnosis	Invasive sampling, Patient discomfort, Unable to reflect entire organ	(120, 121)
	Intestinal mucosal permeability testing	Biochemical analyzer, Liquid chromatography	Non-invasive, High patient compliance, Rapid for large-scale screening, Simple to operate, Suitable for mass screening.	Lack spatial resolution, Low specificity, Susceptible to interference	(30)
	Tight junction protein detection	Western Blot, ELISA Reader	High target specificity, High sensitivity for molecular quantification	Invasive sampling, High cost	(122)
Indirect detection	Bacteriologic examination	Mass spectrometer, Microbiological incubator, PCR instrument	Gold standard for bacterial infections and dysbiosis	Time-consuming, Limited to cultivable bacteria	(123)
	Endotoxin and antibody testing	Endotoxin detector, ELISA reader, Flow Cytometer	Non-invasive sampling, Simple operation and rapid detection.	Low specificity, Cannot localize injury, Antibody window period	(35)
	Infection-related biomarkers testing	Biochemical analyzer, Immunoassay analyzer, Spectrophotometer	Non-invasive sampling, Simple operation and rapid testing, Suitable for critical care	Cannot pinpoint injury location, Low sensitivity may yield false negatives	(124)

## 5 Therapeutic framework from a multi-system perspective

### 5.1 Digestive system

Intestinal mucosal barrier injury increases intestinal mucosal permeability, disrupts the gut microbiota, and facilitates the translocation of bacteria and pathogen-associated molecular patterns (PAMPs). All these can reach the liver and even the entire body through the lymphatic system and portal circulation, causing liver damage, such as inflammatory reactions and fibrosis, and further promoting the progression of liver diseases (38, 39). Conversely, liver injury and cirrhosis, often accompanied by small intestinal bacterial overgrowth (SIBO), oxidative stress, and systemic inflammation, can further compromise intestinal barrier integrity. For example, hypokalemia is a common complication in patients with chronic diseases such as liver cirrhosis. Wu H et al. found that hypokalemia can increase the permeability of the intestinal mucosal barrier, destroy the intestinal barrier, and further lead to bacterial translocation, thereby exacerbating the vicious cycle (40). Additionally, as cirrhosis progresses, the decline in Th17 cells and associated IL-17 production may impair tight junction integrity and reduce antimicrobial peptide secretion, further aggravating intestinal barrier dysfunction (41, 42).

In summary, the intestinal mucosal barrier and the development of liver diseases interact with each other. Consequently, therapeutic strategies aimed at restoring the intestinal barrier hold considerable promise for the management of liver disorders. Among emerging targets, SCFAs (such as acetic acid, propionic acid and butyric acid) may be one of the potential therapeutic targets. Sarkar A et al. found that butyrate can limit the niche of macrophages in non-alcoholic steatohepatitis (NASH), induce the death of pro-inflammatory macrophages to eliminate inflammatory reactions *in vivo* and *in vitro*, and alleviate hepatitis (43). In addition, probiotics also represent a promising therapeutic avenue (44). As a type of intestinal probiotics, *Lactobacillus acidophilus* can improve cholestatic liver injury by inhibiting bile acid synthesis and promoting bile acid excretion (45). It can also inhibit the development of non-alcoholic fatty liver-related hepatocellular carcinoma by producing valeric acid to reduce steatosis and necroinflammation (46). These findings underscore the importance of further exploring intestinal barrier-related mechanisms as a source of novel therapeutic targets for liver diseases.

### 5.2 Urinary system

There is also a close bidirectional communication between the intestine and the kidney, which is vividly referred to as the “gut-kidney axis” (47). As an important component, the destruction of the intestinal mucosal barrier can lead to intestinal flora disorder, intestinal ischemia–reperfusion injury, and deficiency of intestinal nutrients. This will cause inflammatory reactions, further affect metabolism and immunity, causing occurrence and development of kidney diseases (48). Conversely, the accumulation of uremic toxins can also damage the integrity of the intestinal mucosal barrier, further aggravating the original kidney diseases (49). In chronic kidney disease (CKD), for example, indoxyl sulfate (IS) activates the AhR–IRF1 pathway, suppresses DRP1 expression, impairs mitophagy, and ultimately induces intestinal barrier damage (50). Similarly, IgA nephropathy destroys the mucosal barrier by reducing the expression of tight junction proteins. Permitting leakage of intestinal endotoxins and metabolites into circulation. This triggers systemic inflammation that aggravates renal injury, while intestinal B-cell overactivation and excessive IgA production further drive disease progression (49).

Given this pathophysiology, various treatment methods targeting the intestinal mucosal barrier are expected to improve kidney diseases, and relevant studies have also proved the feasibility of this idea, such as probiotics (51) and fecal microbiota transplantation (52). For instance, Meng Y et al. found that Bifidobacterium can effectively improve renal function in AKI mice by regulating intestinal flora imbalance, inhibiting intestinal inflammation and reconstructing the intestinal mucosal barrier. It is worth mentioning that probiotic intervention reversed the adverse consequences of flora imbalance and increased the abundance of potential beneficial bacteria such as Bifidobacterium and Faecalibacterium (51). In another innovative approach, a study developed a hyaluronic acid (HA) nano-coated *Clostridium butyricum*, which exerts anti-inflammatory and tissue repair effects in a cisplatin-induced acute kidney injury (AKI) model through a dual-function and dual-site intervention strategy, with a better effect than the clinical drug NAC (53). In the future, with the deepening of research on the regulatory mechanism of the intestinal mucosal barrier, intervention strategies targeting the barrier are expected to play a greater role in the prevention and treatment of kidney diseases, holding substantial promise for future clinical translation.

### 5.3 Nervous system

The gut and brain communicate bidirectionally through neural, immune, and metabolic pathways. The neural pathway involves the autonomic nervous system and vagus nerve. In the immune pathway, the gut microbiota influences the innate immune system. The metabolic pathway exerts its effects through neurotransmitters, SCFAs, and other factors (54, 55). Within this network, the intestinal mucosal barrier plays an indispensable role. Once the integrity of the barrier is compromised, the intestinal microbiota becomes disrupted. The signaling molecules that control the pathways from the intestinal lumen to the lamina propria (which contains immune cells and ENS neuron terminals) or the portal venous circulation will be affected, causing neurological or psychiatric disorders such as traumatic brain injury, autism spectrum disorder, and depression (7, 54, 56, 57). For example, a high-fructose diet–induced impairment of the intestinal barrier can trigger hippocampal neuroinflammation and neuronal loss (58). Conversely, neurological conditions can also disrupt intestinal barrier integrity, establishing a vicious cycle. Wang H et al. reported that ischemic stroke activates the sympathetic nervous system, which downregulates intestinal Toll-like receptor 5 (TLR5), promoting colonization by flagellated bacteria, damaging the mucus layer, and significantly increasing intestinal permeability (59).

In recent years, these findings have provided potential therapeutic targets for targeting the gut-brain bidirectional communication axis, protecting the intestinal barrier, and improving the prognosis of neurological disorders. These therapeutic approaches include probiotics and prebiotics, FMT, and dietary interventions. Among these, probiotics (such as Bifidobacterium and Lactobacillus) and prebiotics (such as fructooligosaccharides) have demonstrated positive effects in alleviating symptoms of various neurological disorders, and their combined use may enhance efficacy (55). Notably, certain probiotics can also assist in preventing ischemic stroke through the gut-spleen-brain axis (60). Their metabolite, SCFAs, can activate the NLRP6 inflammasome in the colon of mice. This improve hippocampal neuroinflammation, and open up a new direction for the intervention of neurodegenerative diseases (58). A growing number of studies further support intestinal barrier repair as a viable strategy for neurological conditions (61, 62). As mechanistic insights into the gut–brain axis deepen, such targeted approaches are expected to broaden the clinical prospects for preventing and treating neurological disorders.

### 5.4 Respiratory system

The respiratory tract and the intestinal tract share a common mucosal immune system. Although there is no direct anatomical connection between them, they communicate indirectly through the lung-gut axis, which maintains the body’s immune balance and health (63). Among these, the intestine, as the largest bacterial reservoir in the human body (20), plays a key role through its mucosal barrier, which is essential for maintaining homeostasis in the intestine and throughout the body. Once the structure of the intestinal mucosal barrier is damaged, bacteria in the intestine migrate to the lungs due to increased intestinal permeability and mesenteric lymphatic migration, thereby inducing lung diseases such as COPD (64). Conversely, Tao L et al. found that in COPD, inhibition of aryl hydrocarbon receptor (AhR) disrupts the intestinal epithelial barrier and induces intestinal mucosal barrier injury by activating NF-κB (65). Further supporting this bidirectional loop, Ziaka M et al. found that in bronchoalveolar lavage fluid (BALF) and plasma from ARDS patients, IL-1β, TNF-*α*, IL-6, and IL-8 were significantly elevated. Overactivated inflammatory cytokines downregulate tight junction proteins, leading to intestinal hyperpermeability and ultimately intestinal barrier dysfunction, forming a vicious cycle (66).

A comprehensive analysis of the regulatory mechanisms of the lung-gut axis reveals that the intestinal mucosal barrier serves as a therapeutic target for intervening in various lung diseases. Key therapeutic strategies include probiotics, prebiotics, SCFAs, FMT, TCM, and antimicrobial peptides. Probiotics enhance intestinal barrier function by producing SCFAs and upregulating tight junction proteins (67), thereby alleviating COPD symptoms and improving lung function (68). Additionally, Tao L et al. found that supplementing with intestinal antimicrobial peptides (such as lysozyme) can regulate the intestinal microbiota, repair the intestinal mucosal barrier, and reduce high-oxygen-induced lung injury. This suggests that intestinal antimicrobial peptides may serve as potential therapeutic targets for lung injury repair, offering clinical management implications for conditions like bronchopulmonary dysplasia in preterm infants (65).

### 5.5 Integumentary system

The normal function of the intestinal barrier is the first line of defense against harmful pathogens. It is crucial for maintaining the stability of the intestinal environment and the health of skin tissue. When the integrity of the intestinal barrier is compromised, on one hand, allergens, microorganisms, and toxins stimulate the submucosal immune system, releasing cytokines and inflammatory mediators that progressively degrade the intestinal epithelial barrier, creating a vicious cycle. On the other hand, these substances can translocate into the circulatory system, reaching target tissues including the skin, inducing Th2 cells to migrate to inflammatory sites and release inflammatory factors, thereby triggering tissue damage (69). Research has shown that various skin diseases such as atopic dermatitis, psoriasis, and acne are associated with mucosal barrier disruption (70). Additionally, skin inflammation and damage can also reciprocally disrupt the integrity of the mucosal barrier (71, 72). Some studies have pointed out that intestinal microbiota dysbiosis following skin burns is one of the key factors leading to mucus barrier damage. Pathogenic bacteria may degrade mucus through pathways such as glycosidase hydrolysis, flagella, and endotoxins, thereby disrupting the mucosal barrier (73).

**Figure 3:**
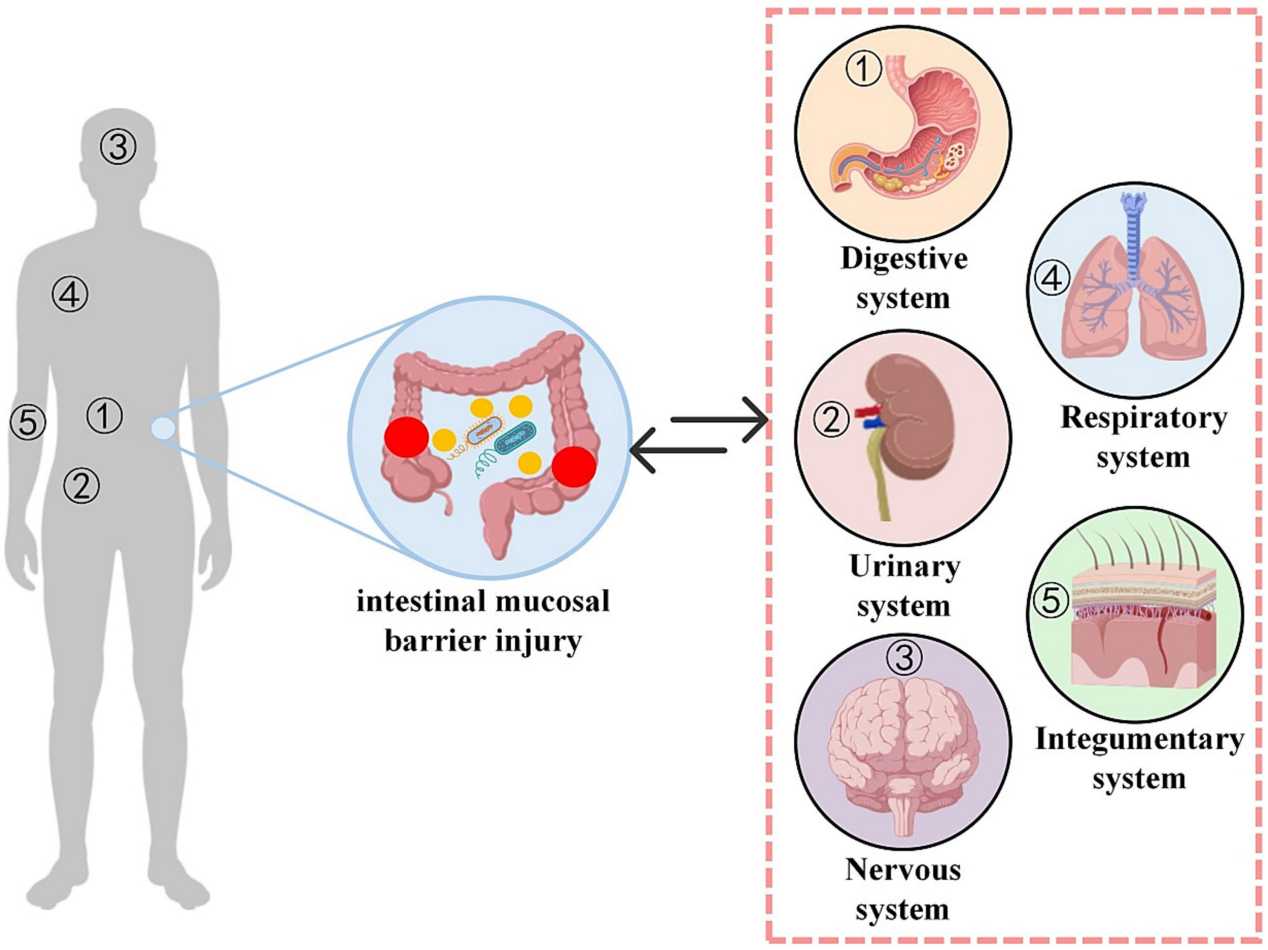
Therapeutic framework of intestinal mucosal barrier injury from a multi-system perspective. This diagram illustrates the mutual interactions between intestinal mucosal barrier injury and multiple systems of the human body. The numbers on the left side of the picture correspond to the representative organs of each system on the right side. 1 represents digestive system; 2 represents urinary system; 3 represents nervous system; 4 represents respiratory system; and 5 represents integumentary system.

Given this interplay, the intestinal mucosal barrier represents a promising therapeutic target for skin diseases. Currently, relevant therapeutic targets primarily focus on probiotics, prebiotics, and TCM. Among these, probiotics play a role in improving intestinal barrier function, restoring intestinal microbiota health, stimulating the host immune system, and counteracting inflammation. They demonstrate significant potential in the prevention and treatment of skin diseases. Studies have shown that probiotics such as lactobacilli and bifidobacteria can provide energy to intestinal epithelial cells under stress through short-chain fatty acids (especially butyrate), promote mucin synthesis, and accelerate the repair of damaged intestinal mucus barriers (73). In the future, with the continuous deepening of research on the mechanisms underlying the gut-skin axis, intervention strategies targeting the intestinal mucosal barrier are expected to open up broader prospects for the clinical treatment of skin diseases. The illustrations corresponding to the content in Sections 4 can be found in Figure 3.

## 6 Research status from a multi-disciplinary perspective

### 6.1 Traditional Chinese medicine

Traditional Chinese medicine, a unique traditional medicine in China, boasts the features of being inexpensive, convenient, and time-tested. TCM intervention has emerged as a promising strategy for intestinal mucosal barrier damage by regulating intestinal microecology and improving dysbiosis (74). Shaowei Huang et al. found that *Paeonia lactiflora* soup could restore intestinal barrier dysfunction by promoting the interaction between group 3 innate lymphoid cells (ILC3) and gut microbiota (75). Furthermore, astragalus polysaccharides (76), ginseng polysaccharides (77), Ganoderma lucidum polysaccharides (78), and baicalein (79) can also improve the diversity of the intestinal microbiota and regulate polyunsaturated fatty acid metabolism to restore intestinal mucosal barrier damage.

These TCM components work through multi-targeted mechanisms. They not only modulate the balance of beneficial and harmful bacteria, but also regulate immune responses and metabolic pathways, creating a favorable environment for the repair of the intestinal mucosal barrier. However, it should be noted that most current evidence comes from preclinical studies, and the precise molecular mechanisms, particularly how specific components target host signaling or microbiota interactions, require further elucidation. Despite these limitations, the holistic regulatory capabilities of TCM provide valuable insights for developing integrated therapeutic strategies against intestinal mucosal barrier injury.

### 6.2 Nutrition

Nutritional support is one of the most significant means of treating intestinal mucosal barrier damage, including enteral and parenteral nutrition. This treatment effectively maintains the normal metabolism and function of intestinal mucosal epithelial cells. It promotes the repair and regeneration of the intestinal mucosa by administering adequate nutrients (80). Common nutritional preparations, such as Glutamine (81, 82), arginine (83), omega-3 polyunsaturated fatty acids (PUFAs) (84), can be applied individually or in various combinations (85).

Beyond these conventional supplements, several key micronutrients demonstrate significant therapeutic potential. Xiaomeng Sun et al. demonstrated that the vitamin D receptor is involved in IBD (86). Further extending this finding, Yu Chen et al. reported that vitamin D derivatives attenuate endotoxemia, alleviate oxidative stress, and suppress inflammatory responses, ultimately reducing early mortality following severe burns and markedly improving intestinal barrier integrity (87). Further extending this finding, Feng C et al. showed that vitamin B12 could effectively improve intestinal epithelial cell injury by regulating the HIF-1 signaling pathway and the intestinal microbiota (88). Feng Y et al. also confirmed that zinc glutathione alleviates alcohol-induced intestinal damage in mice by regulating intestinal zinc transport proteins and enhancing mucosal barrier function (89).

In summary, rational nutritional interventions show promise in preventing and repairing intestinal mucosal damage. However, the field faces challenges including limited clinical translation, incomplete mechanistic understanding, and insufficient attention to individualized nutritional strategies. Future research should prioritize well designed clinical trials to establish protocols and explore synergistic effects of combined nutritional approaches.

### 6.3 Environmental science

Environmental science plays a crucial role in damage to the intestinal mucosal barrier. With the acceleration of industrialization, environmental pollution problems have become increasingly serious. In particular, the impact of environmental factors such as heavy metals (90), pesticide residues (91), and air pollution (17, 92) on intestinal health cannot be ignored.

Occupational exposures present another dimension of risk, though translational relevance to human populations requires careful interpretation. In a landmark space environmental study, Jiang P et al. conducted fecal sequencing on mice after 37 days of flight on the International Space Station and combined it with liver transcriptome analysis. They found that spaceflight increased the diversity of *α*-intestinal microorganisms and altered the community structure (93), revealing that the special microenvironment in space may cause damage to the intestinal mucosal barrier. This results lay a foundation for in-depth understanding of the physiological impact of the space environment on astronauts and provide an important research direction for ensuring the health of astronauts during space exploration. Similarly, Liu X et al. discovered that low-dose ionizing radiation can significantly alter the composition and function of the intestinal microbiota in mice, manifested as reduced diversity, decreased beneficial bacteria, increased opportunistic pathogens, and metabolic imbalance (94). These changes may also induce damage to the intestinal mucosal barrier. This study has important practical implications for radiation-related professionals, such as radiologists and nuclear industry workers, providing new scientific evidence for their occupational health protection.

In summary, while environmental and occupational factors undoubtedly influence intestinal barrier integrity, current understanding is constrained by limited human studies and mechanistic data. In the future, it should be given high priority, and measures such as environmental governance and optimization of occupational protection should be strengthened. At the same time, due to differences in lifestyle habits and occupational exposure among individuals, the manifestations of barrier injury may also vary. Therefore, it is necessary to fully consider individual differences and develop personalized plans to more effectively protect intestinal health.

### 6.4 Psychology

The integrity of the intestinal mucosal barrier is crucial for maintaining intestinal health. In recent years, the impact of psychological factors on the function of the intestinal mucosal barrier has gradually attracted attention. Long-term psychological stress and anxiety can lead to dysfunction of the intestinal mucosal barrier, which in turn triggers intestinal inflammation and dysregulation of the intestinal microbiota (95). Shaler CR et al. found that stress leads to a significant increase in inflammatory cytokines such as TNF and IFN-*γ*. They also discovered that a decrease in the expression of genes related to barrier function (such as mucin genes, tight junction genes, etc.) in the ileum, and an increase in ileal permeability. These lead to bacterial translocation and triggering systemic inflammatory responses (96). Moreover, psychological stress may also affect the intestinal microbiota indirectly through the neuroendocrine system, influencing the integrity of the intestinal mucosal barrier (97).

Consequently, psychological interventions and stress management are increasingly considered valuable components of intestinal barrier protection strategies. Approaches such as psychotherapy, relaxation training, and mindfulness meditation show promise in mitigating psychological stress and potentially supporting barrier function. However, robust clinical evidence demonstrating their efficacy specifically in restoring intestinal barrier integrity remains limited, highlighting the need for well-designed intervention studies in relevant patient populations.

### 6.5 Genetics

Genetics plays a crucial role in intestinal mucosal barrier injury. Its influence spanning the entire process of barrier function maintenance, susceptibility to damage, and repair. Wang S et al. found that homozygous deletion of the Myh9 gene in the intestinal epithelium of adult mice leads to colitis-like morphological changes. It also disrupts the integrity of the intestinal lumen barrier, and promotes necroptosis of epithelial cells. Furthermore, the deletion of this gene also results in a reduction in the number of stem cells, increases the sensitivity of mice to dextran sulfate sodium, and accelerates the formation of colitis-associated adenomas in the colon. These findings reveal that variations in the Myh9 gene are closely associated with intestinal mucosal barrier function and related diseases (98). In addition, Wang S et al. discovered that the intra-villous microadenomas formed after heterozygous deletion of the APC gene are the root cause of intestinal mucosal polyps in the APC delta 716 type. This result confirms that APC gene mutations can lead to abnormal intestinal mucosal structure (99). Moreover, variations in the lactase gene (LCT) can affect the abundance of Bifidobacteria (100, 101). As an important probiotic, the reduction in the number of Bifidobacteria can also exacerbate intestinal mucosal barrier damage to a certain extent.

Notably, studies have indicated that applying systems genetics (multi-omics) approaches to research on the human genome and intestinal microbiome, through integrating data from gene expression, proteomics, metabolomics, and other omics levels. This will be an inevitable path for in-depth analysis of the complex ecological interactions between the human host and microorganisms in the future (102).

However, while these platforms offer unprecedented analytical depth, their translation into clinically actionable insights remains challenging due to the polygenic nature of barrier disorders and substantial interindividual heterogeneity. Their direct application in personalized barrier protection strategies will require validation in diverse human populations and deeper functional characterization of identified genetic variants.

### 6.6 Food science

Food science provides an important basis for maintaining intestinal health by exploring the interactions between food components, processing technologies, and the intestinal mucosal barrier. Abundant nutrients (103), plant compounds (104), probiotics (105, 106) and other components in food can play a role in protecting the intestinal mucosal barrier through multiple mechanisms. Among them, probiotics (such as Bifidobacteria and Lactobacilli) can enhance barrier function by competitively inhibiting the adhesion of pathogenic bacteria to the intestinal mucosa, secreting antibacterial substances and stimulating intestinal epithelial cells to secrete mucus and tight junction proteins (107). Prebiotics (such as oligosaccharides) can provide nutritional support for probiotics, indirectly helping to maintain barrier health (67, 107).

While modern food processing technologies enhance palatability and extend shelf life, their potential impact on the intestinal mucosal barrier warrants critical attention. A growing body of evidence suggests that a diet rich in ultra-processed foods (UPFs) is associated with intestinal diseases, including inflammatory bowel disease, colorectal cancer, and irritable bowel syndrome. Many components in UPFs, such as emulsifiers, sweeteners, colorants, microparticles, and nanoparticles, can have adverse effects on the intestinal microbiota, intestinal permeability, and intestinal inflammatory status. This may also interfere with the normal function of the intestinal mucosal barrier (108).

**Figure 4:**
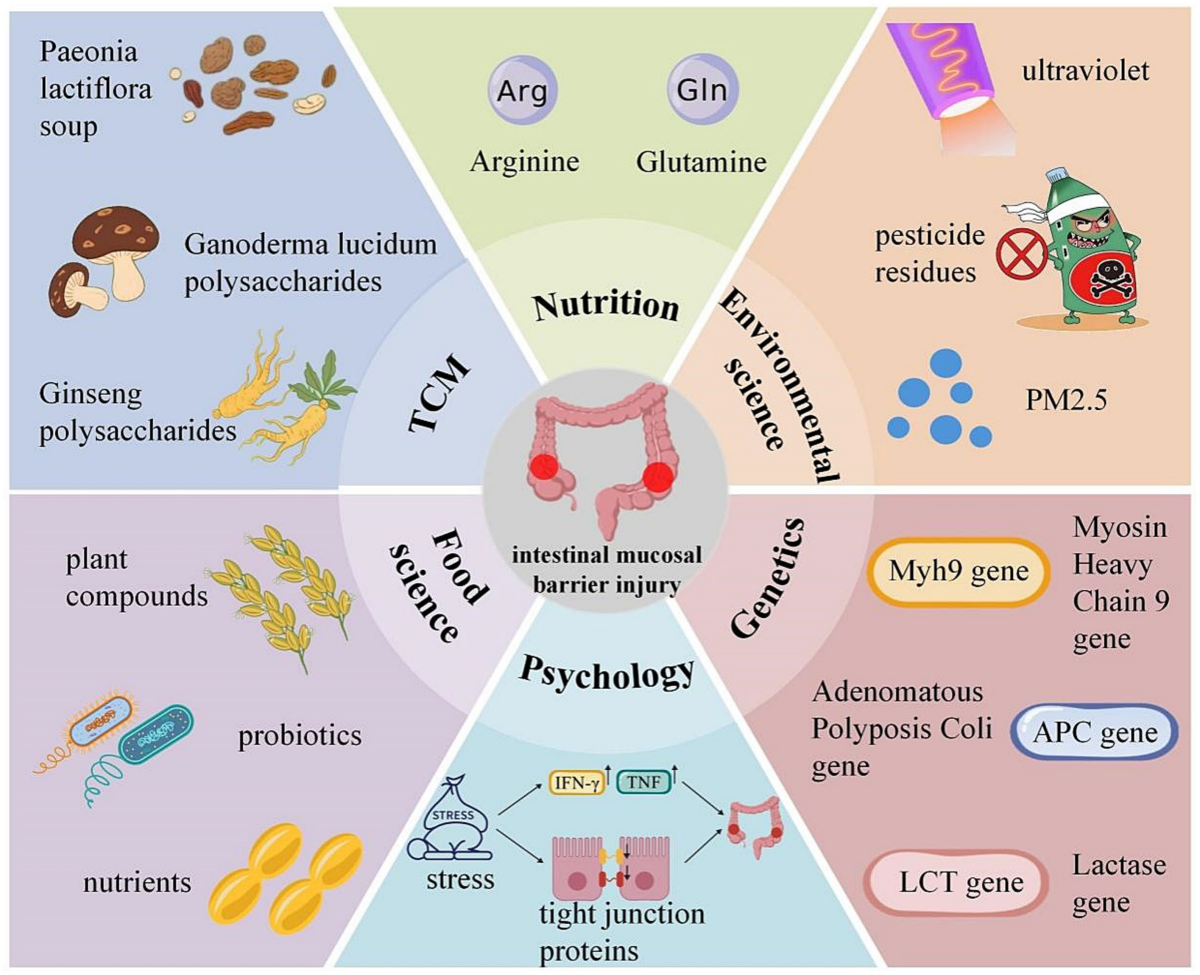
Research status on intestinal mucosal barrier injury from a multi-disciplinary perspective. The figure shows various factors affecting intestinal mucosal barrier injury from multiple perspectives such as Traditional Chinese Medicine (TCM) (e.g., *Paeonia lactiflora* soup, Ganoderma lucidum polysaccharides, Ginseng polysaccharides), Nutrition (e.g., Arginine, Glutamine), Environmental science (e.g., ultraviolet, pesticide residues, PM2.5), Genetics (e.g., Myh9 gene, APC gene, LCT gene), Psychology (e.g., stress), and Food science (e.g., plant compounds, probiotics, nutrients). TCM, Traditional Chinese Medicine; Myh9 gene, Myosin Heavy Chain 9 gene; APC gene, Adenomatous Polyposis Coli gene; LCT gene, Lactase gene.

In the future, with the in-depth exploration of food-intestine interaction mechanisms and the optimization of food formulas and processing technologies, an important path will emerge. This path will help improve intestinal health and reduce the risk of intestinal diseases. The illustrations corresponding to the content in Sections 5 can be found in Figure 4.

## 7 Prospects for intestinal mucosal barrier injury

With the rapid development of modern medicine, the study of intestinal mucosal barrier injury will usher in a broader prospect. In the future, the research of intestinal mucosal barrier injury will pay more attention to the following aspects:

### 7.1 Exploring the molecular mechanisms of intestinal mucosal barrier injury

Through advanced technical means such as molecular biology and cell biology, the molecular mechanism of intestinal mucosal barrier injury can be explored in depth. Revealing the essential laws of its occurrence and development will provide more accurate and reliable theoretical support for the prevention and treatment of intestinal mucosal barrier injury. For example, some studies have shown that by regulating the MCs/Tryptase/PAR - 2/MLCK pathway, the intestinal mucosal injury of rats with diarrhea-type irritable bowel syndrome can be protected (109). Additionally, research efforts have focused on exploring the cyclic adenosine 3′, 5′-monophosphate (cAMP) signaling pathway for the treatment of intestinal mucosal barrier injury (110). Based on the study of these detailed molecular mechanisms, researchers develop personalized therapeutic approaches. These approaches fully leverage the potential of macrophages in maintaining and restoring intestinal permeability and safeguarding intestinal health (111, 112).

### 7.2 Using new tests and therapeutic drugs

With the continuous progress of science and technology, more sensitive, accurate and convenient tests and therapeutic drugs will be developed in the future. They will provide more powerful support for clinical treatment. Cutting-edge endoscopic imaging technologies, such as ultra-high-magnification endoscopy and probe-based confocal laser endoscopy, are new technologies capable of exploring the “cellular” intestinal injury in real time. In addition, new advanced spatial imaging platforms have emerged. These include multispectral imaging, upconverted nanoparticles, digital spatial analysis, spectroscopic and mass spectrometric flow cytometry. Such technologies enable in-depth and comprehensive assessment of the gut barrier at the molecular and ultrastructural levels (112, 113).

### 7.3 Exploring the application of TCM in intestinal mucosal barrier protection

Chinese medicine has unique advantages in intestinal mucosal barrier protection. In the future, the application of TCM in intestinal mucosal barrier protection will be promoted. This promotion aims to explore its more specific protective mechanisms and combined therapeutic effects, thereby providing new ideas and methods for the prevention and treatment of intestinal mucosal barrier damage. For example, a study has shown that epigallocatechin (EC) and *β*-glucan (BG) in whole barley grains can alleviate high-fat diet-induced intestinal barrier dysfunction and regulate intestinal flora dysbiosis in mice (114). This finding provides new perspectives and directions for TCM-related research.

### 7.4 Conducting multidisciplinary research

Intestinal mucosal barrier injury involves multiple disciplinary fields, such as basic medicine (115), clinical medicine, and pharmacology. In the future, strengthening multidisciplinary cross-research is of paramount importance. This endeavor requires promoting communication and cooperation among various disciplines to collectively advance the research on intestinal mucosal barrier injury. For example, helminths parasitizing the intestine are in close contact with the mucosal surface and cause a series of local immune changes affecting the intestinal barrier layer. Helminths are seen as a potential factor in the prevention and even treatment of many diseases associated with epithelial barrier theory. Expanded intestinal epithelial stem cells *in vitro*, obtained directly from mucosal biopsies or through targeted differentiation from human pluripotent stem cells, hold promise as a complementary therapeutic option for mucosal injury patients. This potential stems from their pluripotency and ability to differentiate into all epithelial cell types of the gut (116, 117). Nevertheless, transplantation of bone marrow mesenchymal stem cells (BMSCs) has demonstrated favorable therapeutic effects in the treatment of a variety of diseases caused by ischemia or reperfusion injury (118, 119).

## 8 Conclusion

Intestinal mucosal barrier injury is a complex condition that extends its impact well beyond the gastrointestinal system, influencing multiple organ systems and contributing to the pathogenesis of a variety of diseases. The intestinal barrier plays a crucial role in maintaining both local and systemic health through its mechanical, chemical, biological, and immune components. Disruption of this barrier is linked to diverse diseases such as IBD, liver cirrhosis, obesity, diabetes, and even neurological conditions, highlighting its far-reaching effects on overall health.

The multi-system impact of intestinal mucosal barrier injury underscores the importance of a multidisciplinary, integrated approach to research and treatment. Mechanisms such as microbial dysbiosis, oxidative stress, ischemia–reperfusion injury, and inflammatory responses contribute to the breakdown of the intestinal barrier, leading to increased intestinal permeability and translocation of harmful substances into the bloodstream. These factors not only disrupt the gastrointestinal system but also initiate or exacerbate systemic inflammation, affecting distant organs like the liver, brain, kidneys, and skin. This interconnectedness of systems calls for a holistic view, recognizing that damage to the intestinal barrier often triggers a cascade of pathological events across multiple body systems.

Emerging research across fields such as microbiology, immunology, nutrition, and environmental science has provided significant insights into the complex relationship between intestinal mucosal injury and systemic diseases. For example, the gut-liver axis and gut-brain axis demonstrate how intestinal barrier dysfunction can influence the progression of liver diseases and neurological disorders. Furthermore, the interaction between gut microbiota and immune responses plays a pivotal role in the development of systemic inflammatory conditions, emphasizing the need for therapies that target both the gut and other organ systems.

## Glossary

AKI: Acute Kidney Injury
APC gene: Adenomatous Polyposis Coli gene
ARDS: Acute Respiratory Distress Syndrome
BALF: Bronchoalveolar Lavage Fluid
BG: β-glucan
BMSCs: Bone Marrow Mesenchymal Stem Cells
cAMP: Cyclic Adenosine 3′, 5’-Monophosphate
CKD: Chronic Kidney Disease
COPD: Chronic Obstructive Pulmonary Disease
DCs: Dendritic Cells
DRP1: Dynamin-Related Protein 1
EC: Epigallocatechin
ENS: Enteric Nervous System
FMT: Fecal Microbiota Transplantation
GALT: Gut-Associated Lymphoid Tissue
HA: Hyaluronic Acid
HIF-1: Hypoxia-Inducible Factor 1
IBD: Inflammatory Bowel Disease
IFN-γ: Interferon-γ
IgA: Immunoglobulin A
IL-17: Interleukin-17
IL-1β: Interleukin-1β
IL-8: Interleukin-8
ILC3: Group 3 Innate Lymphoid Cells
IS: Indoxyl Sulfate
LCT: Lactase gene
MCs: Mast Cells
MLCK: Myosin Light Chain Kinase
Myh9 gene: Myosin Heavy Chain 9 gene
NAC: N-Acetylcysteine
NASH: Non-Alcoholic Steatohepatitis
NF-κB: Nuclear Factor Kappa B
NLRP6: NLR Family Pyrin Domain Containing 6
NOX1-NOS2: NADPH Oxidase 1-Nitric Oxide Synthase 2
NSAIDs: Non-Steroidal Anti-Inflammatory Drugs
PUFA: Omega-3 Polyunsaturated Fatty Acids
PAMPs: Pathogen-Associated Molecular Patterns
PAR – 2: Protease-Activated Receptor-2
ROS: Reactive Oxygen Species
SCFAs: Short-Chain Fatty Acids
sTREM-1: Soluble Myeloid Expression-triggered Receptor
TCM: Traditional Chinese Medicine
TLR5: Toll-like Receptor 5
TNF-α: Tumor Necrosis Factor-a
UPFs: Ultra-Processed Foods
ZO1: Zonula Occludens-1
ZO3: Zonula Occludens-3
